# What Do We Know Now about IgE-Mediated Wheat Allergy in Children?

**DOI:** 10.3390/nu9010035

**Published:** 2017-01-04

**Authors:** Grażyna Czaja-Bulsa, Michał Bulsa

**Affiliations:** 1 Paediatrics and Paediatric Nursery Unit, Pomeranian Medical University, Żołnierska 48, Szczecin 71-210, Poland; 2 Division of Paediatrics, Gastroenterology and Rheumatology of the “Zdroje” Hospital, Szczecin 70-780, Poland; 3 Chair and Department of Pathology, Pomeranian Medical University, Unii Lubelskiej 1, Szczecin 71-252, Poland; michal.bulsa@gmail.com

**Keywords:** wheat allergy, specific immunoglobulin E, children, gluten-related disorders

## Abstract

IgE-mediated wheat allergy is a gluten-related disorder. Wheat is one of the five most common food allergens in children. However, the natural history of IgE-mediated wheat allergy has seldom been described in the research literature. This study presents the current state of knowledge about the IgE-mediated wheat allergy in children.

## 1. Introduction

Wild wheat grains (*Triticum aestivum*) were consumed by people in America as early as 11,000 years BC (before Christ). Table 1 shows the centuries of utilization of wheat by people.

Today almost a half of the calories consumed by the human population worldwide come from cereals, with wheat being the most popular grain in Europe and the Americas. Its use is so widespread that people suffering from gluten-related disorders have great difficulty in avoiding it. Flour and bran are used in the production of bread, muesli, breakfast cereals, pasta, bulgur, couscous, and pastries. Being a binding agent, wheat is added to cold cuts, desserts, ice cream, and cream. Starch is used for coating pills, pralines, and roasted coffee grains, as well as in cosmetic, paper, and chemical industries.

## 2. Wheat-Related Allergic Disorders

**Figure 1:**
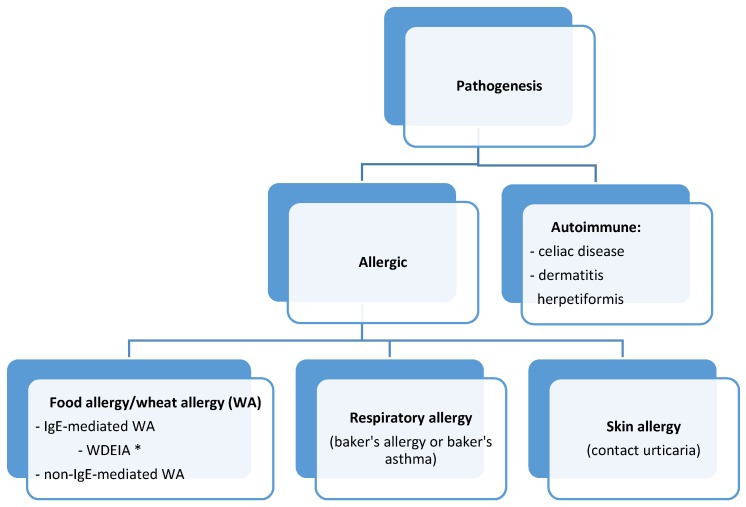
Classification of wheat-related allergic diseases. * WDEIA—wheat-dependent exercise-induced anaphylaxis.

Depending on the routes of entry, wheat-related allergic disorders are classified into: food allergies, respiratory allergies, and skin allergies (Figure 1) [1]. The allergy classifications also consider celiac disease (CD) as a wheat-related allergic disorder. However, CD is, rather, an autoimmune disease and in most countries it is treated according to gastroenterological protocols. Food allergies triggered by wheat consumption are divided to IgE-dependent wheat allergy (WA) and IgE-non-dependent WA [1]. One of the WA syndromes is also wheat-dependent, exercise-induced anaphylaxis (WDEIA). In the latest EAACI (European Academy of Allergy and Clinical Immunology) classification it has been recognized as an independent form of wheat allergy [1].

**Figure 2:**
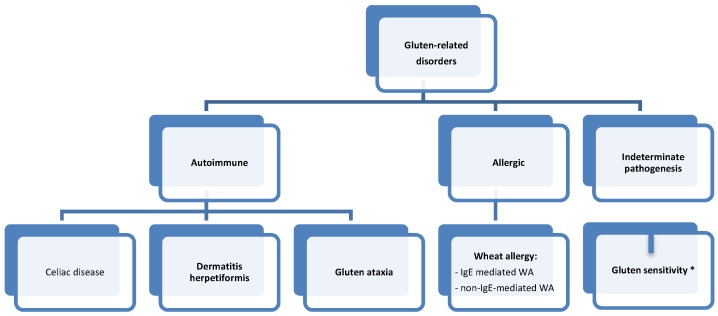
Classification of gluten-related disorders. * Gluten sensitivity (GS) = non-celiac gluten sensitivity (NCGS).

WA is also one of the gluten-related disorders, the classification of which was published in 2012 (Figure 2) [2]. It is important to note that although WA belongs to this group, all of its forms stem solely from the adverse effect of wheat proteins (including gluten proteins). Therefore, the treatment is based on the elimination of wheat grains only. The allergy induced by proteins contained in other gluten grains is less common and has not been included in the classification discussed herein.

## 3. Prevalence of IgE-Mediated Wheat Allergy

Wheat is one of the five most common foods that trigger allergic reactions in children. In Germany, Japan, and Finland it has been reported as the third most common allergen, after milk and egg [3]. WA prevalence, both in children and adults, is usually approximately 1% (0.4%–4%), depending on age and region [3,4,5,6]. In patients with food allergies, WA is diagnosed in 11%–20% of children and in 25% of adults [7]. Taking into account all gluten-related disorders, it has been estimated that about 3% of the human population suffers from wheat intolerance (1% WA, 1% celiac disease, 1% non-celiac gluten sensitivity) [4,5,8].

## 4. Clinical Picture of IgE-Mediated Wheat Allergy

WA prevails chiefly in children with a family history of atopy. Almost all of the juvenile WA patients are diagnosed with allergies to other foods and other allergic disorders, most commonly atopic dermatitis (78%–87%). Half of patients suffer from asthma (48%–67%) and/or allergic rhinitis (34%–62%) [9,10]. The majority of children are allergic to cow’s milk (80%), chicken egg white (56%–72%), fish (28%), soya (24%–50%), and peanuts (29%–50%) [9,10,11,12,13].

The WA clinical picture depends on age [10]. Symptoms develop within minutes to 1–2 h after the ingestion of wheat. In young children gastroenterological symptoms prevail, such as vomiting, diarrhea or, rarely, abdomen pains. In about 40% of children skin symptoms are observed in a form of urticaria, erythema, angioedema, pruritus, or worsening atopic dermatitis [9,10,12,13]. Intestinal symptoms recede with age; therefore, older children suffer mostly from dermatitis, which is accompanied by respiratory disorders (wheeze, stridor, persistent cough, hoarse voice, respiratory distress, nasal congestion) and, in the most severe cases, anaphylaxis. In teenagers and adults the most severe forms of allergy prevail, such as anaphylaxis symptoms (in 45%–50%), which is typical of wheat allergy. Intestinal and skin symptoms are less common in these age groups [1,9,10,12].

WA is usually diagnosed in young children, but it is rarely seen in infants, despite the fact that wheat proteins pass into breast milk, which was proven by Linn et al. in 1996 [14]. In our study on 50 children with WA, the disease was diagnosed in 32% of infants. Three of them were fed exclusively on their mother’s milk [10]. In 1981, Rudd et al. described a case of an infant with anaphylactic shock after consuming semolina pudding [15].

WA can be accompanied by allergies to other cereals, most often to rye and/or corn [12].

## 5. Development of Tolerance to IgE-Mediated Wheat Allergy

The prognosis of WA tolerance is not as poor as it is in the case of allergies to peanuts, shellfish, or fish that usually continue into adulthood. It is similar to the tolerance prognosis in children allergic to milk or egg. WA will prevail to maturity in about 10% of patients, with the most severe clinical forms of the disorder [9,10,11]. In our study the age median of the tolerance was seven years (3–16 years). Fifty-two percent of patients developed the tolerance by the age of eight years, 66% of them by the age of 12, and 76% by the age of 16 [10]. Other researchers have reported similar results [11].

Similarly to other food allergies, the progress of tolerance can be assessed by means of the wheat IgE titers determined repeatedly during the elimination diet. When wheat IgE concentration increases, tolerance is unlikely to develop quickly. Conversely, it is likely to occur when the wheat IgE titers decline steadily. Moreover, such a procedure allows the determination of the maximum wheat IgE concentration. The higher the titers, the older the patient at the time when the tolerance develops. In our studies the tolerance age median was 3.5 years when the maximum wheat IgE concentrations were below 19.9 kU/L. When it was within the range of 20–49.9 kU/L, the median rose to seven years. It reached 16 years of age when the concentrations exceeded 50 kU/L [10].

## 6. Wheat-Dependent, Exercise-Induced Anaphylaxis—A Rare Form of Wheat Allergy

WDEIA is a rare syndrome [16]. It is typically diagnosed in adults and sporadically in older children. It is clinically characterized by anaphylactic reactions (hives, Quincke’s edema, shock) occurring 10–60 min after exercise following the ingestion of wheat 10 min to four hours earlier. The amount of ingested wheat and the intensity of exercise can vary substantially. The rω-5 gliadin is the major WDEIA allergen—it is found in all the patients. The serum concentrations of rω-5 gliadin-specific IgE are correlated with the severity of the WDEIA clinical response. IgE serum concentrations for the rω-5 gliadin higher than 0.89 kU/L confirm the WDEIA diagnosis (sensitivity 78%, specificity 96%), while the sIgE sensitivity for whole wheat extract and gluten is low (48% and 56%, respectively) [17].

## 7. Wheat Grain Proteins

All of the wheat-induced diseases are caused by wheat proteins which constitute 10%–18% of the grain mass, depending on the strain. The main component (70%) of the wheat grain is starch.

Depending on their dissolving agent, the wheat grain proteins are categorized into four main fractions: albumins (15%), globulins (7%), gliadins (33%), and glutenins (45%). Albumins are soluble in water; globulins, in salt solutions; gliadins, in alcohol; and while glutenins, in dilute acid and alkali. Albumins and globulins are structural proteins that contain many enzymes. Gliadins and glutenins are prolamins and are referred to as gluten. They are storage proteins.

The wheat proteins that are regarded today as the WA major allergens will be discussed in detail in the next chapter. Wheat proteins triggering CD symptoms belong to gliadins. Wheat proteins responsible for non-celiac-gluten sensitivity (NCGS) have not been identified yet. One of the proteins under the researchers’ examination is a group of amylase-trypsin inhibitors (ATIs) that do not belong to glutens (JunkerY et al., 2012) [18].

## 8. Major Allergens of IgE-Mediated Wheat Allergy

In the serum of patients with IgE-mediated wheat allergy numerous IgE antibodies are found that bind with proteins of all of the wheat grain fractions, most commonly with gliadins. However, in different examinations, they are not the same proteins and, therefore, they cannot be regarded as the major allergens in children with WA [19,20]. A protein can be regarded as the major allergen when IgE antibodies specific for this protein are found in a considerable number of children with WA.

The list of the World Health Organization includes 27 wheat allergens [21]. Clinical relevance of many of them has not been determined yet.

The best-understood allergenic molecule of WA is rω-5 gliadin (Tri a 19) [16,22,23]. The rω-5 gliadin-specific IgEs are present in all patients with WDEIA, in 80% of children with anaphylaxis symptoms after wheat ingestion, and in 20%–30% of children with WA and atopic eczema [20,24,25,26,27,28].

The second allergenic molecule of WA, for which commercial tests are available, is a non-specific lipid transfer protein (Tri a 14) (nsLTP). Antibodies signaling the presence of the IgE specific to Tri a 14 are found in WA children and in patients with WDEIA. They are not very sensitive. It is now thought that they do not exhibit cross-reactivity with grass pollen, although there is not enough data to exclude this. Their assessment may help in differentiating wheat sensitization from pollen allergy, which is vital in patients with high levels of grass pollen-specific IgE [1].

In everyday allergological practice the possibility of swift and simple exclusion of cross-reactions is very important. Wheat is highly cross-reactive with other cereals; mainly rye and barley [20]. It has been shown that prolamins, like gamma-70 and gamma-35 secalins in rye, as well as gamma-3 hordein in barley, cross-react with rω-5 gliadin [29]. These three cereals contain several other proteins that are highly cross-reactive. It has also been confirmed that there is high sequence identity (>80%) among many other proteins, such as alpha-purothionins from wheat, rye, and barley [30]. Positive SPTs and elevated assays of IgE specific to whole wheat extract are common among atopic patients. Up to 65% of the patients with grass pollen allergy have false positive results when tested for wheat extract, i.e., they do not report any health problems after the ingestion of wheat [31]. If the medical history of patients with positive SPT results and with IgE specific to whole wheat extract does not rule out negative reactions to wheat, it is necessary to perform a wheat challenge test, which is rather time consuming. Such a test will be positive in barely 20% of the patients and such a low probability of positive allergy incidence in allergic individuals is reported only in WA.

Today, intense studies are conducted on some wheat protein components: glutenins with low and high molecular weight (LMW-glutenins and HMW-glutenins), α-, β-, and γ-gliadins, as well as non-specific lipid transport protein Tri a 14 [32,33]. However, none have reached a high specificity and sensitivity to become a gold standard in the diagnosis of WA and, therefore, the precise diagnosis still relies on standardized challenges which must be done under medical supervision [1,31,33].

## 9. Diagnosis of IgE-Mediated Wheat Allergy

The WA diagnosis is difficult because not all of the major wheat grain allergens are recognized. Similarly, to any other allergy, the gold standard of WA diagnosis remains the oral food challenge. It is usually performed in its open form, as the majority of the observed adverse reactions is of the objective nature. The patient is given whole wheat starting from small doses of wheat-specific protein (1–50 mg) followed by increasingly larger hourly doses (digestion of wheat can be slower than egg or milk), ending with a cumulative dose of up to 0.5–1 g of wheat protein [1]. Additionally, double-blind placebo-controlled protocols of WA have been published both for children and adults [28,34]. WA is diagnosed when the challenge test results are positive and the symptoms appear up to two hours after ingestion.

In the next stage, allergological tests should be performed to confirm the elevated levels of wheat allergen-specific IgE. The first are skin tests (SPTs) to wheat flour. Generally, commercial wheat extract is used, the specificity of which is very low [1]. Some allergologists prepare an in-house wheat flour solution, but its specificity is also very low. It can be improved by additional testing to ω-5 gliadin or other gliadins, but these solutions are not routinely available and are mainly used in scientific research.

Another step is the determination of serum concentrations of allergen-specific IgE to whole wheat extract. They are commercially available but their specificity is low despite high sensitivity [1,33].

Solutions used for skin tests to wheat flour and for the assessment of allergen-specific IgE to whole wheat extract consist of the mixture of grain albumins and globulins and, thus, do not contain the insoluble major wheat allergens, i.e., prolamins. This is why the utility of these tests in WA diagnosis is lower than in allergies to other foods, such as milk, egg, or peanuts [35]. Moreover, their concentration is not correlated with the severity of clinical reactions after wheat ingestion.

Gluten-specific IgE can also be assessed. Since the commercial test contains wheat gluten proteins, it is positive only in the case of a wheat allergy and negative in the case of allergies to other gluten-containing cereals. It is not known if it includes major wheat allergens. Gluten-specific IgE assays are positive in two thirds of children with WA [10].

Currently, there are commercial tests for the IgE specific to two known allergenic molecules of wheat: Tri a 14 non-specific lipid transfer protein and Tri a 19 rω-5-gliadin [1]. Their importance to the WA diagnosis has been discussed in the section “Major Allergens in IgE-Mediated Wheat Allergy” of this paper.

It is characteristic of children who had WA, and have developed a tolerance to wheat, that in most of them (approximately 80%) SPTs to wheat continue to be positive and IgE specific to whole wheat extract and gluten-specific IgE remain elevated, which is rare in other food allergies [9,10]. For the majority of food allergens, tolerance development is accompanied by negative SPTs and normalized specific IgE levels. In our studies, at the time of tolerance development, the levels of IgE specific to whole wheat extract ranged between 0.35–23.9 kU/L (median: 3.0 kU/L) [10]. In that group of patients, at the time of WA diagnosis, the levels of IgE specific to whole wheat extract had been between 2.2 kU/L and 39.3 kU/L (median: 8.42 kU/L). This is why the size of SPT and the levels of IgE specific to whole wheat extract are not useful in differentiating between the periods of allergy and tolerance. It should be stressed, however, that in individual children who have developed tolerance to wheat the SPT values and IgE specific to whole wheat extract and gluten are lower than when WA was diagnosed.

## 10. Treatment of IgE-Mediated Wheat Allergy

It is worth emphasizing that even though IgE-mediated wheat allergy belongs to gluten-dependent disorders, it is induced solely by wheat proteins, thus being treated by a wheat-free diet. The remaining gluten cereals, such as rye, barley, and oats are well tolerated by most patients and should not be eliminated from their diet. In WA children, oat allergy is very rare. Rye and barley allergies are slightly more common.

It is believed that different species of wheat have the same allergenicity, therefore, it is not recommended for the patients with severe WA to try different forms of wheat. There are no studies describing changes in allergenicity of wheat during processing [1].

Moreover, it is not recommended to routinely administer a gluten-free diet to WA patients. Gluten-free products made from rice or corn flour, tapioca, millet, or sorghum, are usually well tolerated by WA patients. However, such products often contain wheat starch as the main ingredient which can be insufficiently purified of wheat proteins to be safe for WA patients. The gluten-free products dedicated to CD patients can contain no more than 20 mg of gluten proteins per 1 kg.

Since 2009 the packaging of all products sold in the European Union must inform consumers about the wheat content.

In 2013 the case was reported of two children with anaphylaxis after wheat ingestion who had developed prior tolerance to pressure-cooked whole wheat. The process of pressure-cooking changes the structure of the wheat husk. It is not known, however, if it damages the structure of the allergens [36]. No other cases of such a two-stage process of tolerance to wheat have been reported so far.

The research literature has provided the first report on the oral immunotherapy administered to older children with anaphylaxis triggered by wheat ingestion. After two years, the therapy resulted in the desensitization in 61% of the patients [37]. Further studies are necessary to evaluate the effectiveness of this type of WA treatment [1].

In the USA the rω-5 gliadin-free variety of wheat has been produced [38].

## 11. Prophylaxis against IgE-Mediated Wheat Allergy

It is presently believed that the time when foods are introduced into the infants’ diet is relevant to their tolerance. For half of the century, from 1955 to 2005, Europe, America, and Australia used nutrition charts for infants where the delayed introduction of strong allergens was strictly recommended. It is a general opinion that those schemes were responsible for the increased incidence of allergies in that period of time. Today, it is recommended to introduce foods containing strong allergens into the infants’ diets as early as between the 17th and 26th weeks of life.

The initial exposure to wheat grains delayed until after six months of age may increase the risk of wheat allergy [39]. So far only one prospective study has been published (EAT study) which assesses the effects of introducing wheat (and other allergens) after the third vs. the sixth month of life on the incidence of WA in three year olds. The study was inconclusive because in neither group had WA been diagnosed [40].

In recent years the results of two extensive studies (Celiprev, Prevent CD) have been published that indicate that the time of introducing wheat into the diet of infants at high risk of celiac disease has no effect on the CD prevalence [41,42]. In spring 2016, ESPGHAN issued recommendations to introduce wheat into the infant diet between the 4th and 12th months of life.

## Figures and Tables

**Table 1:** History of wheat cultivation and consumption.

Years	Event
11,000 years BC	Wild wheat grains were consumed by people in America.
7800 years BC	The first records of wheat cultivation began in fertile lands of Southwest (Palestine) and Middle Asia (Mesopotamia); people living in farming settlements grew wheat and barley.
400 years BC	The type of wheat that could be used for baking bread or pastries was first cultivated in China.
100 years BC	The first bread prepared with the use of the brewer’s yeast was baked in France.
